# Aortic remodeling after thoracic endovascular aortic repair and Provisional ExTension To Induce COmplete ATtachment to treat complicated type B aortic dissection

**DOI:** 10.1016/j.jvscit.2026.102363

**Published:** 2026-06-23

**Authors:** Ga-Young Suh, Johan Bondesson, Shanmugesh Raja, Keith Naqvi, Christopher P. Cheng, Brant W. Ullery

**Affiliations:** a Department of Biomedical Engineering, California State University, Long Beach, CA; b Department of Surgery, Stanford University, Stanford, CA; c Oregon Vascular Specialists, Portland, OR

**Keywords:** Dissection, TEVAR, PETTICOAT, Remodeling, Computed tomography

## Abstract

**Objective:**

Aortic remodeling after thoracic endovascular aortic repair (TEVAR) for complicated type B aortic dissection remains insufficiently characterized, particularly when the Provisional ExTension To Induce COmplete ATtachment (PETTICOAT) technique is applied. This study evaluated early geometric remodeling of the true and false lumens and the whole aorta following TEVAR with PETTICOAT using patient-specific computational modeling.

**Methods:**

A single-center retrospective review of 20 patients (16 men; age, 65 ± 11 years; nine acute, seven subacute, and four chronic) with type B aortic dissection treated with TEVAR and PETTICOAT was performed. Pre- and postintervention computed tomography angiography was used to extract centerlines and cross-sectional measurements along the entire aorta. Patient-specific computational simulations quantified changes in aortic arclength, curvature, cross-sectional area, and circumference across the ascending aorta, arch, descending thoracic aorta, and abdominal aorta. Remodeling was assessed by comparing geometric parameters from preoperative and the first available postoperative computed tomography angiography (mean interval from surgery, 82 ± 305 days; median, 14 days).

**Results:**

In the first 30 days, one patient died, and the rest were free from reintervention. False lumen thrombosis was documented in 10 patients (50%) during early (<30 day) imaging and in 14 patients (70%) within 24-month surveillance. TEVAR with PETTICOAT produced significant true lumen expansion, including reduced curvature in the aortic arch and descending thoracic aorta (*P* < .05), and increased abdominal aortic arclength (13.1 ± 2.6 to 13.8 ± 2.3 cm; *P* < .05). True-lumen circumference increased at the arch (10.1 ± 1.7 to 10.5 ± 1.4 cm), descending thoracic aorta (7.1 ± 1.2 to 9.1 ± 1.2 cm), and abdominal aorta (5.7 ± 1.0 to 6.5 ± 1.0 cm; *P* < .05). The whole aorta circumference increased only in the descending thoracic segment (*P* < .05), whereas the false lumen cross-sectional area showed no significant change.

**Conclusions:**

TEVAR with the PETTICOAT technique results in measurable geometric remodeling in the first 30 days, most pronounced within the true lumen across thoracic and abdominal segments. Whole aorta and false lumen changes were limited, suggesting a preferential true lumen response to intervention.

Aortic dissection is a pathologic condition characterized by a tear of the intimal layer of the aortic wall, leading to the separation of the wall layers in the thoracic aorta and the potential for distal propagation into the aortoiliac segment and branch vessels. Aortic dissection occurs in 4 to 14 per 100,000 persons/year worldwide.^1^ It is a life-threatening disease when left untreated because the weakened aortic wall is at increased risk for rupture and circulatory collapse. In addition, antegrade blood flow directed between the layers of the aortic wall, referred to as the false lumen, may result in malperfusion to the kidneys, bowel, lower extremities, or spinal cord.^1^ Approximately 30% to 40% of aortic dissections do not involve the ascending aorta and are referred to as type B aortic dissections (TBAD). Treatment of TBAD is commonly performed using endovascular stent grafts, a procedure referred to as thoracic endovascular aortic repair (TEVAR), with the primary goals of covering the proximal entry tear, expanding the true lumen, and depressurizing the false lumen.^2^ Although TEVAR has been shown to accomplish these goals in the treated segment of the thoracic aorta, multiple studies have cited high rates of aortic reintervention and undesired expansion of the distal aorta below the endograft, resulting in false lumen aneurysmal degeneration and risk for delayed aortic rupture.^3^, ^4^, ^5^, ^6^

Provisional ExTension To Induce COmplete ATtachment (PETTICOAT) technique is a hybrid approach combining conventional TEVAR (to cover the proximal entry tear) and placement of a bare stent distal to the endograft (to expand the collapsed true lumen in the distal thoracoabdominal segment with branch vessels). This technique is believed to facilitate total aortic remodeling and resolve malperfusion by sealing the distal re-entry tears with a bare metal stent. In particular, the bare metal stent can serve as a scaffold to keep the true lumen expanded without blocking the critical branch vessels in cases of acute complicated TBAD characterized by branch vessel malperfusion.^7^ The Study of Thoracic Aortic Type B Dissection Using Endoluminal Repair I trial reported 100% technical success at 30 days and 90% overall survival at 1 year.^7^ Study of Thoracic Aortic Type B Dissection Using Endoluminal Repair II demonstrated 100% technical success at 30 days with a 12.3% secondary intervention rate at 1 year, and 97.1% freedom from dissection-related mortality maintained through 5 years.^8^^,^^9^ Several studies demonstrated that PETTICOAT techniques are effective in reducing aortic-related adverse events, not only for acute and complicated type B dissections, but also for chronic dissections.^10^, ^11^, ^12^

Despite these promising outcomes, there are concerns related to coverage, device durability, and long-term performance that persist.^13^^,^^14^ The mixed outcomes emphasize the need for careful planning of TEVAR with the PETTICOAT technique and assessment of aortic remodeling during surveillance.^15^ Accurate evaluation of the aortic remodeling is achievable with geometric analysis of the thoracic aorta and abdominal aorta, tracking both true and false lumina. The objective of the present study is to quantify localized geometric changes after intervention to examine early outcomes of TEVAR with the PETTICOAT technique on remodeling of the thoracic and abdominal aorta. This was achieved through three-dimensional computational modeling based on patient-specific computed tomography (CT) data, systematically extracting geometric features of the aorta before and after TEVAR with the PETTICOAT technique. Previous studies examining aortic remodeling after TEVAR and PETTICOAT have predominantly reported volumetric or maximum diameter changes at discrete anatomic levels. The present study extends this by applying a continuous, segmental geometric analysis that captures curvature, arclength, circumference, and cross-sectional area along the entire thoracoabdominal aorta, enabling spatial mapping of remodeling that distinguishes true lumen from whole aorta responses.

## Methods

### Subject recruitment and CT data collection

This retrospective, image-based study was approved by the local institutional review board with waiver of informed consent. Patients undergoing TEVAR with the PETTICOAT technique to treat complicated TBAD between 2019 and 2024 were included in this study. According to the Society for Vascular Surgery/Society of Thoracic Surgeons Reporting Standards for TBADs, entry tear at zone 1+ was classified as TBAD, with distal extent ranging from zone 5 (distal thoracic aorta above the celiac trunk) to zone 11 (below iliac bifurcation).^16^ Complicated TBAD was defined as cases with clinical and/or radiographic malperfusion, aortic diameter exceeding 40 mm at index presentation, rapid aortic growth (ie, >5 mm during the first hospital admission), intractable pain, or refractory hypertension.^10^ Cases not meeting these criteria were designated uncomplicated TBAD and excluded from this study. Additional radiographic exclusion criteria included the following (1) pre- or post-TEVAR CT missing or acquired without contrast, (2) image slice thickness greater than 2 mm, (3) visible imaging artifact along the thoracic aorta, and (4) insufficient field of view (eg, cropped thoracic ascending aorta). The PETTICOAT technique was used at the surgeon’s discretion in patients meeting the above criteria for complicated TBAD. Postoperative imaging followed the institutional surveillance protocol: CT acquisition at 1 , 6 , and 12 months, and annually thereafter. The first available contrast-enhanced postoperative CT was used for geometric analysis. All CT data included in this study were acquired by a single-source CT scanner (LightSpeed VCT, GE) or a dual-source CT scanner (SOMATOM Definition, Siemens).

### Computational modeling from CT data

**Fig 1:**
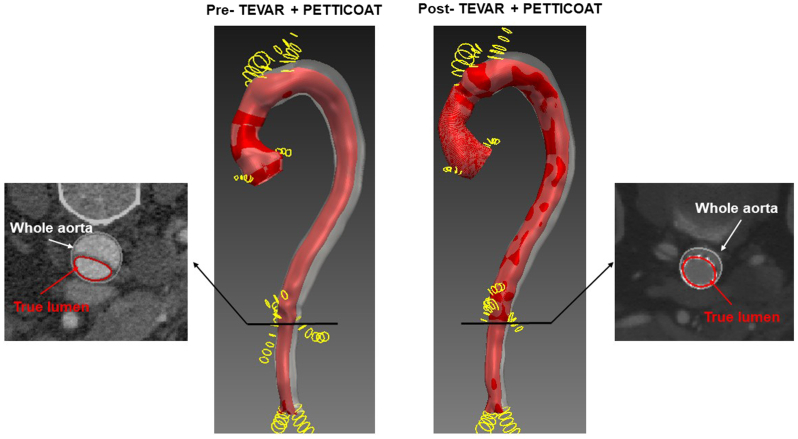
Definition of true lumen and the whole aorta during the segmentation.

Three-dimensional geometric models, including lumen centerline and cross-sectional contours of the thoracic aorta and endograft, were constructed using open-source software (SimVascular, Open Source Medical Software Corporation).^17^^,^^18^ The vessels of interest included the thoracic aorta (such as ascending, arch, and descending), abdominal aorta, and branch vessels, including the coronary arteries, brachiocephalic artery, left subclavian artery, celiac artery, and common iliac arteries. The aorta was segmented with two separate contours: (1) the aortic true lumen (the lumen bounded by the dissected flap and the nondissected aortic wall) and (2) the whole aorta (the aorta bounded by the adventitia layer). Due to its complex anatomical nature, this study used manual segmentation by selecting points along the perimeter of the lumen of interest (separately for the true lumen and whole aorta) and automatically connecting them with a closed spline to form a closed contour.^18^, ^19^, ^20^ The lumen bound by the metallic struts from the endograft or bare stent was accounted as the true lumen, and connected with the unrepaired dissected true lumen (Fig 1). The sampling interval along the aortic length to extract the cross-sectional contours was set at 5 to 10 mm (approximately half of the lumen radius), with a wider interval used where the lumen size was larger.^21^

**Fig 2:**
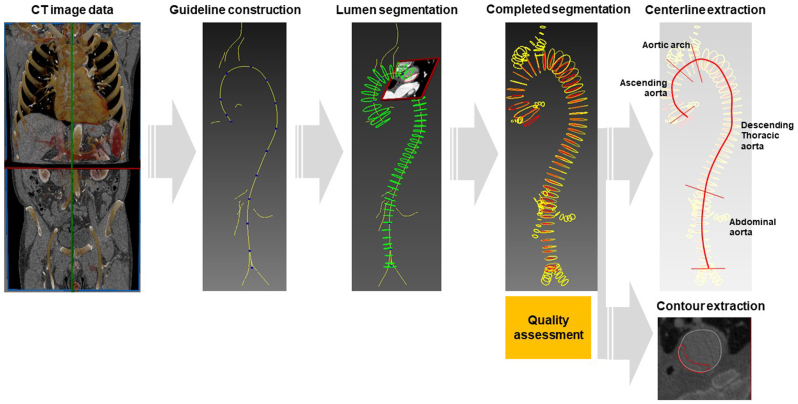
Workflow for computed tomography (*CT*)-based geometric modeling.

The left coronary and brachiocephalic branches were used to define longitudinally the ascending thoracic aorta, the brachiocephalic and left subclavian branch locations defined the aortic arch, the left subclavian and celiac branch locations defined the descending thoracic aorta, and the celiac branch and aortic bifurcation defined the abdominal aorta. Post-TEVAR, the true lumen included the endograft + distal bare stent, which naturally followed the true lumen boundaries. The quality of the paths and segmented contours was assessed by a second operator who was not engaged in modeling to avoid bias. After segmentation was completed, centerlines representing the true lumen and the whole aorta were automatically extracted^22^ (Fig 2).

### Geometric feature extraction

Longitudinal metrics, including the arclength and curvature, were quantified using the aortic centerline for each aortic section: ascending thoracic aorta, aortic arch, descending thoracic aorta, and abdominal aorta. Arc length was calculated as the axial length of the centerline. Curvature was calculated as the inverse of the radius of a circumscribed circle fitted onto three evenly spaced points on the centerline.^21^^,^^22^ After the curvature at every 1 mm was quantified, the mean curvature was calculated as an average within each aortic section. In addition, the max curvature within each aortic section was recorded.

Cross-sectional metrics, including lumen circumference and cross-sectional area, were quantified using the cross-sectional lumen contours for each aortic section. The circumference was the arclength of the perimeter of the two-dimensional lumen contour.^23^ The cross-sectional area was calculated as the area bounded by the lumen contour.^24^ For both features, the mean values were calculated as the average within each aortic section. Since the whole aorta and true lumen were segmented separately, all geometric features were extracted independently for the whole aorta and true lumen. The false lumen was characterized by its cross-sectional area, which was the difference between the area of the whole aorta and the area of the true lumen. All geometric features were computed automatically using custom MATLAB scripts (MathWorks). A summary of all geometric features, their definitions, and the aortic sections to which they were applied is provided in Table I.

**Table I:** Geometric features and their definitions for corresponding aortic sections

Geometric feature, unit	Definition	Aortic boundary	Aortic section
Arc length, cm	Axial length of the aortic centerline within the aortic section	True lumen	Ascending thoracic aorta, aortic arch, descending thoracic aorta, and abdominal aorta
		Whole aorta	Ascending thoracic aorta, aortic arch, descending thoracic aorta, and abdominal aorta
Mean curvature, /cm	Curvature averaged within the aortic section (curvature is defined as the inverse of the radius of a circumscribed circle fitted along the aortic centerline)	True lumen	Ascending thoracic aorta, aortic arch, descending thoracic aorta, and abdominal aorta
		Whole aorta	Ascending thoracic aorta, aortic arch, descending thoracic aorta, and abdominal aorta
Max curvature, /cm	Local maximum curvature within the aortic section	True lumen	Ascending thoracic aorta, aortic arch, descending thoracic aorta, and abdominal aorta
		Whole aorta	Ascending thoracic aorta, aortic arch, descending thoracic aorta, and abdominal aorta
Circumference, cm	Arclength of the perimeter of the two-dimensional lumen contour defining the aortic boundary	True lumen	Ascending thoracic aorta, aortic arch, descending thoracic aorta, and abdominal aorta
		Whole aorta	Ascending thoracic aorta, aortic arch, descending thoracic aorta, and abdominal aorta
Cross-sectional area, cm^2^	Lumen area within the aortic boundary	True lumen	Ascending thoracic aorta, aortic arch, descending thoracic aorta, and abdominal aorta
		Whole aorta	Ascending thoracic aorta, aortic arch, descending thoracic aorta, and abdominal aorta
		False lumen	Ascending thoracic aorta, aortic arch, descending thoracic aorta, and abdominal aorta

True lumen is the lumen bounded by the dissected flap and the nondissected aortic wall. The whole aorta is the aorta bounded by the adventitia layer. The false lumen is the difference between the whole aorta and the true lumen.

### Statistical analysis

Paired, two-tailed *t*-tests were used to compare metrics at different states (pre- vs post-TEVAR values) with a statistical significance threshold set at a *P* value of .05. The *t*-tests were performed after ensuring the normality of the data using the Shapiro-Wilk test. For non-normally distributed data, the Wilcoxon signed-rank test was used to compare samples. All statistical tests were performed using SPSS version 29 (IBM).

## Results

### Patient demographics and clinical outcomes

This study included 20 patients (16 males; age, 65 ± 11 years) with TBAD treated with TEVAR with PETTICOAT at a single institution. Baseline demographics and patient characteristics are summarized in Table II. Type B dissections were acute in nine patients, subacute in seven patients, and chronic in four patients. The extent of dissection was exclusively thoracoabdominal in four patients and with iliac extension in 16 patients. Based on pre-TEVAR CT review, the maximum aortic diameter was 4.3 ± 1.3 cm at the thoracic aorta and 3.3 ± 0.8 cm at the abdominal aorta. The time interval from pre-TEVAR CT acquisition to the surgery date was 14 ± 21 days. One patient had their pre-TEVAR CTs 67 days before surgery owing to a chronic TBAD with progressive Leriche syndrome requiring delayed surgical intervention, and 19 patients had their pre-TEVAR CTs within 33 days before surgery. Distribution of thoracic aortic endograft manufacturers included seven Zenith TX2 (Cook Medical), four Valiant Captivia (Medtronic), three Zenith Alpha (Cook Medical), three thoracic branch endoprosthesis (W.L. Gore), two TAG Conformable (W.L. Gore), and one Valiant Navion (Medtronic). Proximal coverage was zone 0 in one patient, zone 2 in 12 patients, and zone 3 in seven patients. Diameter of proximal device was ≥40-mm in three patients, 32 to 38 mm in 15 patients, and 30 to 31 mm in two patients. Thoracic aortic endograft coverage extended into zone 4 in three patients and zone 5 in the remaining 17 patients. The Zenith Dissection Endovascular Stent (Cook Medical) was used exclusively for distal thoracoabdominal aortic coverage (46 mm diameter device, n = 2; 36-mm diameter device, n = 18). Adjunctive procedures included celiac artery percutaneous balloon angioplasty, n = 1; visceral stenting, n = 3; renal artery drug-eluting balloon angioplasty, n = 1; renal artery stenting, n = 2; aortic septotomy, n = 2; iliac stenting, n = 3; and false lumen embolization, n = 1.

**Table II:** Baseline demographics and patient characteristics

Variables	N (%)
Demographics	
Age, years (mean ± SD)	65 ± 11
Gender, male	16 (80)
Body mass index, kg/m^2^ (mean ± SD)	30 ± 6
Comorbidities	
Coronary artery disease	2 (10)
Congestive heart failure	3 (15)
Previous myocardial infarction	1 (5)
Hyperlipidemia	4 (20)
Hypertension	13 (65)
Diabetes mellitus	3 (15)
Chronic renal insufficiency	1 (5)
COPD	3 (15)
Tobacco use	8 (40)
Prior aortic surgery	6 (30)
Acuity	
Acute	9 (45)
Subacute	7 (35)
Chronic	4 (20)

*COPD*, Chronic obstructive pulmonary disease; *SD*, standard deviation.

Categorical data are shown as number (%).

Early post-TEVAR and follow-up outcomes are summarized in Table III. Mean intensive care unit and total hospital length of stay were 2 ± 3 and 4 ± 4 days, respectively. Within 30 days of surgery, one patient developed visceral malperfusion from static obstruction of the celiac origin and required celiac stent placement on postoperative day 13. Despite successful restoration of mesenteric perfusion, this patient expired 18 days after surgery. During follow-up surveillance (>30 days), one patient developed enterococcal bacteremia and a rapidly growing mycotic aneurysm, necessitating reintervention, and this patient expired 1513 days after surgery. Three patients developed type II endoleak. Thoracic aortic false lumen thrombosis was noted in one-half of patients on initial postoperative imaging (<30 days) and in 14 patients within 2 years postoperatively. The time interval from surgery to the post-TEVAR CT ranged from 1 to 1511 days (mean, 82 ± 305 days; median, 14 days), with 16 patients imaged within 40 days, three patients within 50 to 70 days, and one patient at 1511 days. A sensitivity analysis excluding the patient with the 1511-day postoperative CT interval (n = 19) did not materially alter the primary geometric findings; all previously significant comparisons remained statistically significant (*P* < .05), confirming the robustness of results to this outlier. In addition, Spearman correlation analysis revealed no significant association between the postoperative CT imaging interval and the magnitude of geometric change.

**Table III:** Early post-thoracic endovascular aortic repair (*TEVAR*) and follow-up outcomes

Variables	N (%)
Early post-TEVAR outcomes (<30 days)	
Death	1 (5)
Spinal cord ischemia	0 (0)
Malperfusion	1 (5)
Intraoperative endoleak (type IA)	0 (0)
Reintervention	1 (5)
Follow-up outcomes (>30 days)	
Death	1 (5)
Reintervention	4 (20)
Postoperative imaging	
Endoleak (type I)	0 (0)
Endoleak (type II)	3 (15)
Endoleak (type III)	0 (0)
False lumen, patent	5 (25)
False lumen, partially thrombosed	4 (20)
False lumen, thrombosed	10 (50)

### Geometric changes due to TEVAR with PETTICOAT

From pre- to post-TEVAR with the PETTICOAT technique, the true lumen exhibited significant geometric changes. The arc length of the abdominal aorta had increased from 13.1 ± 2.6 to 13.8 ± 2.3 cm (*P* < .05). The mean curvature was significantly reduced at the aortic arch (0.26 ± 0.08 to 0.22 ± 0.07 cm^−1^; *P* < .05), whereas the true lumen mean curvature of the other segments remained similar. The peak curvature was reduced at the aortic arch (0.32 ± 0.10 to 0.27 ± 0.08 cm^−1^; *P* < .05) and descending thoracic aorta (0.34 ± 0.09 to 0.31 ± 0.06 cm^−1^; *P* < .01). Mean circumference was increased at the aortic arch (10.1 ± 1.7 to 10.5 ± 1.4 cm; *P* < .05), descending thoracic aorta (7.1 ± 1.2 to 9.1 ± 1.2 cm; *P* < .001), and abdominal aorta (5.7 ± 1.0 to 6.5 ± 1.0 cm; *P* < .001). The mean cross-sectional area increased from pre- to post-TEVAR with PETTICOAT at the aortic arch (8.3 ± 2.8 to 9.0 ± 2.4 cm^2^; *P* < .05), descending thoracic aorta (3.8 ± 1.6 to 6.8 ± 1.7 cm^2^; *P* < .05), and abdominal aorta (2.4 ± 0.1 to 3.4 ± 1.1 cm^2^; *P* < .05) (Table IV).

**Table IV:** Geometries along the true lumen for pre- and post-thoracic endovascular aortic repair (*TEVAR*) + Provisional ExTension To Induce COmplete ATtachment (*PETTICOAT*) and their comparisons

Geometric feature	Aortic section	Pre-TEVAR + PETTICOAT	Post-TEVAR + PETTICOAT	Post − pre
Arc length, cm	Ascending thoracic aorta	10.8 ± 1.6 (6.5-13.4)	10.3 ± 2.4 (3.9-13.6)	−0.5 ± 1.8 (−7.7 to 0.9)
	Aortic arch	2.7 ± 1.5 (1.3-8.3)	3.1 ± 1.9 (1.0-9.5)	0.4 ± 1.6 (−2.1 to 5.9)
	Descending thoracic aorta	28.7 ± 3.4 (22.4-34.0)	29.2 ± 3.8 (21.9-35.6)	0.5 ± 1.8 (−2.0 to 6.0)
	Abdominal aorta	13.1 ± 2.6^a^ (8.6-18.0)	13.8 ± 2.3^a^ (9.7-17.9)	0.8 ± 1.4 (−2.3 to 4.4)
Mean curvature, /cm	Ascending thoracic aorta	0.21 ± 0.03 (0.17-0.26)	0.20 ± 0.05 (0.09-0.28)	−0.00 ± 0.04 (−0.14 to 0.07)
	Aortic arch	0.26 ± 0.08^a^ (0.07-0.42)	0.22 ± 0.07^a^ (0.06-0.38)	−0.04 ± 0.06 (−0.17 to 0.02)
	Descending thoracic aorta	0.13 ± 0.03 (0.08-0.20)	0.12 ± 0.03 (0.08-0.19)	−0.00 ± 0.01 (−0.02 to 0.03)
	Abdominal aorta	0.12 ± 0.06 (0.05-0.28)	0.11 ± 0.06 (0.04-0.27)	−0.00 ± 0.03 (−0.05 to 0.05)
Max curvature, /cm	Ascending thoracic aorta	0.34 ± 0.05 (0.23-0.42)	0.34 ± 0.11 (0.13-0.66)	−0.00 ± 0.10 (−0.26 to 0.26)
	Aortic arch	0.32 ± 0.10^a^ (0.10-0.51)	0.27 ± 0.08^a^ (0.09-0.41)	−0.06 ± 0.08 (−0.20 to 0.07)
	Descending thoracic aorta	0.34 ± 0.09^a^ (0.22-0.51)	0.31 ± 0.06^a^ (0.21-0.42)	−0.04 ± 0.05 (−0.15 to 0.02)
	Abdominal aorta	0.22 ± 0.12 (0.09-0.47)	0.22 ± 0.13 (0.08-0.55)	0.00 ± 0.07 (−0.19 to 0.12)
Circumference, cm	Ascending thoracic aorta	11.6 ± 1.8 (7.8-14.6)	11.4 ± 1.3 (9.0-14.5)	−0.2 ± 1.3 (−4.6 to 2.4)
	Aortic arch	10.1 ± 1.7^a^ (7.2-13.4)	10.5 ± 1.4^a^ (8.2-13.2)	0.4 ± 0.9 (−0.8 to 2.5)
	Descending thoracic aorta	7.1 ± 1.2^a^ (5.4-10.1)	9.1 ± 1.2^a^ (6.9-10.9)	2.0 ± 1.1 (0.6-3.9)
	Abdominal aorta	5.7 ± 1.0^a^ (4.1-7.3)	6.5 ± 1.0^a^ (5.1-8.8)	0.8 ± 0.5 (−0.1 to 1.7)
Cross-sectional area, cm^2^	Ascending thoracic aorta	10.8 ± 3.3 (4.7-17.2)	10.2 ± 2.4 (6.3-16.5)	−0.6 ± 2.5 (−9.4 to 4.1)
	Aortic arch	8.3 ± 2.8^a^ (3.9-14.1)	9.0 ± 2.4^a^ (5.3-14.0)	0.7 ± 1.4 (−1.3 to 3.9)
	Descending thoracic aorta	3.8 ± 1.6^a^ (1.8-8.3)	6.8 ± 1.7^a^ (3.8-9.4)	3.0 ± 1.6 (0.8-6.0)
	Abdominal aorta	2.4 ± 1.0^a^ (1.0-4.3)	3.4 ± 1.1^a^ (1.8-6.3)	0.9 ± 0.7 (−0.1 to 2.9)

Values are mean ± standard deviation (minimum-maximum).

a Indicates the pair with *P* value <.05 from the comparison between pre- and post-TEVAR + PETTICOAT geometries.

**Fig 3:**
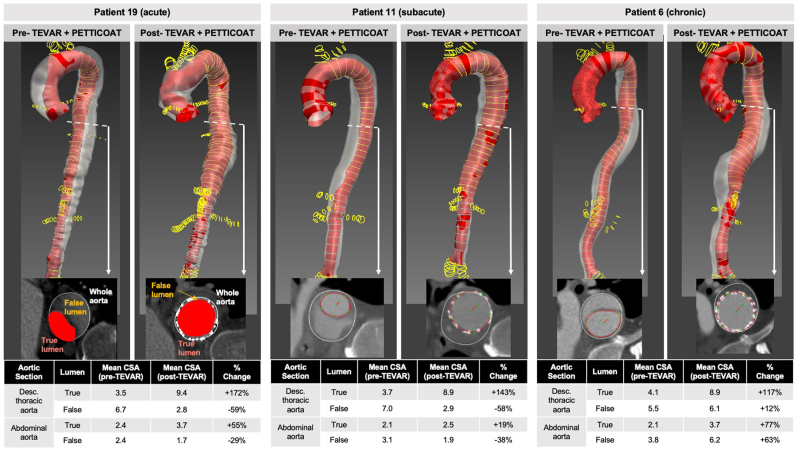
Representative geometric changes from pre- to post-thoracic endovascular aortic repair (*TEVAR*) with the Provisional ExTension To Induce COmplete ATtachment (*PETTICOAT*) from acute, subacute, and chronic patients. The true lumen (*red*) and whole aorta (*gray*) are rendered separately. The axial cross-sectional image shows the midlevel of the descending thoracic aorta where the endograft was implanted. The mean cross-sectional area (*CSA*, in cm^2^) of the true and false lumens at the descending thoracic and abdominal aortic segments before and after intervention, with percentage change, is presented for each patient in the *bottom* row.

The geometric changes of the whole aorta were less notable than for the true lumen. There was no significant change in the whole aorta arclength or curvature from pre- to post-TEVAR with PETTICOAT. The mean circumference was significantly increased at the descending thoracic aorta (11.3 ± 1.5 to 12.4 ± 2.2 cm; *P* < .05). Similarly, the cross-sectional area was increased at the descending thoracic aorta (10.4 ± 2.8 to 12.6 ± 4.5 cm^2^; *P* < .05) (Table V). Despite significant changes in the cross-sectional area of the true lumen and the whole aorta, the area of the false lumen did not exhibit a significant change from pre- to post-TEVAR with PETTICOAT (Table VI). No significant difference in geometric change was noted based on age or gender. Moreover, subgroup analysis by dissection acuity [acute (n = 9), subacute (n = 7), and chronic (n = 4)] revealed no statistically significant between-group differences in any geometric outcome (all *P* > .05). Fig 3 illustrates the example case from each group, showing geometric changes from pre- to post-TEVAR with PETTICOAT.

**Table V:** Geometries along the whole aorta for pre- and post-thoracic endovascular aortic repair (TEVAR) + Provisional ExTension To Induce COmplete ATtachment (PETTICOAT) and their comparisons

Geometric feature	Aortic section	Pre-TEVAR + PETTICOAT	Post-TEVAR + PETTICOAT	Post - pre
Arclength, cm	Ascending thoracic aorta	10.7 ± 1.6 (6.6-13.4)	10.3 ± 2.3 (4.0-13.6)	−0.5 ± 1.8 (−7.5 to 0.9)
	Aortic arch	2.8 ± 1.5 (1.3-8.2)	3.1 ± 1.9 (1.0-9.5)	0.3 ± 1.5 (−1.9 to 5.8)
	Descending thoracic aorta	28.8 ± 3.5 (22.3-34.1)	29.2 ± 3.7 (22.1-35.5)	0.4 ± 1.8 (−2.3 to 5.9)
	Abdominal aorta	14.9 ± 2.6 (11.9-20.9)	15.1 ± 2.5 (11.8-20.3)	0.2 ± 0.6 (−1.4 to 1.4)
Mean curvature, /cm	Ascending thoracic aorta	0.20 ± 0.03 (0.16-0.24)	0.20 ± 0.04 (0.09-0.28)	0.00 ± 0.03 (−0.10 to 0.08)
	Aortic arch	0.22 ± 0.06 (0.06-0.33)	0.21 ± 0.07 (0.05-0.32)	−0.01 ± 0.04 (−0.10 to 0.05)
	Descending thoracic aorta	0.14 ± 0.03 (0.09-0.20)	0.13 ± 0.03 (0.09-0.19)	−0.00 ± 0.01 (−0.03 to 0.03)
	Abdominal aorta	0.12 ± 0.06 (0.05-0.29)	0.12 ± 0.06 (0.05-0.28)	−0.00 ± 0.02 (−0.04 to 0.06)
Max curvature, /cm	Ascending thoracic aorta	0.32 ± 0.05 (0.22-0.43)	0.31 ± 0.08 (0.14-0.46)	−0.00 ± 0.07 (−0.19 to 0.12)
	Aortic arch	0.28 ± 0.08 (0.09-0.44)	0.25 ± 0.08 (0.07-0.38)	−0.02 ± 0.07 (−0.19 to 0.09)
	Descending thoracic aorta	0.33 ± 0.08 (0.21-0.45)	0.31 ± 0.05 (0.23-0.40)	−0.02 ± 0.08 (−0.16 to 0.15)
	Abdominal aorta	0.23 ± 0.12 (0.07-0.51)	0.23 ± 0.12 (0.09-0.49)	−0.00 ± 0.05 (−0.09 to 0.12)
Circumference, cm	Ascending thoracic aorta	12.0 ± 1.4 (9.9-15.2)	12.2 ± 2.1 (9.3-19.1)	0.2 ± 1.2 (−0.9 to 3.9)
	Aortic arch	10.9 ± 1.3 (9.0-14.7)	11.3 ± 1.6 (9.2-15.2)	0.3 ± 1.0 (−0.8 to 3.2)
	Descending thoracic aorta	11.3 ± 1.5^a^ (8.7-14.4)	12.4 ± 2.2^a^ (9.0-16.8)	1.1 ± 1.8 (−0.6 to 6.5)
	Abdominal aorta	8.4 ± 1.2 (5.8-10.6)	9.1 ± 1.8 (6.5-12.7)	0.7 ± 1.7 (−0.8 to 5.5)
Cross-sectional area, cm^2^	Ascending thoracic aorta	11.5 ± 2.7 (7.6-18.4)	12.1 ± 4.8 (6.8-28.8)	0.5 ± 3.0 (−2.0 to 10.4)
	Aortic arch	9.7 ± 2.5 (6.5-17.4)	10.4 ± 3.1 (6.8-28.8)	0.7 ± 2.0 (−1.6 to 6.3)
	Descending thoracic aorta	10.4 ± 2.8^a^ (6.2-16.8)	12.6 ± 4.5^a^ (6.6-22.6)	2.2 ± 3.9 (−0.9 to 14.0)
	Abdominal aorta	5.7 ± 1.6 (2.7-9.1)	6.8 ± 2.8 (3.4-13.0)	1.1 ± 2.6 (−1.2 to 8.9)

Values are mean ± standard deviation (minimum-maximum).

a Indicates the pair with *P* value <.05 from the comparison between pre- and post-TEVAR + PETTICOAT geometries.

**Table VI:** Geometries along the false lumen for pre- and post-thoracic endovascular aortic repair (TEVAR) + Provisional ExTension To Induce COmplete ATtachment (PETTICOAT) and their comparisons

Geometric feature	Aortic section	Pre-TEVAR + PETTICOAT	Post-TEVAR + PETTICOAT	Post - pre
Cross-sectional area, cm^2^	Ascending thoracic aorta	0.8 ± 1.9 (0.0-6.4)	1.8 ± 5.1 (0.0-21.1)	1.1 ± 4.5 (−0.8 to 20.1)
	Aortic arch	1.7 ± 1.9 (0.0-5.6)	1.5 ± 1.9 (0.0-7.0)	−0.2 ± 2.1 (−3.8 to 4.5)
	Descending thoracic aorta	6.7 ± 2.7 (0.3-11.9)	5.9 ± 4.1 (0.2-15.1)	−0.8 ± 4.1 (−6.3 to 13.5)
	Abdominal aorta	3.3 ± 1.5 (0.0-6.2)	3.5 ± 2.4 (0.7-9.4)	0.1 ± 2.6 (−3.6 to 8.5)

Values are mean ± standard deviation (minimum-maximum).

## Discussion

TEVAR with the PETTICOAT technique results in noticeable geometric changes of the aorta at the aortic arch and thoracoabdominal aorta. The aortic true lumen exhibited more dramatic geometric remodeling than the whole aorta and false lumen in the descending and abdominal aorta. From a pre- to post-TEVAR comparison, the geometric changes in the whole aorta were primarily within the descending thoracic aorta. In contrast, those in the true lumen were more extensive, from the aortic arch to the abdominal aorta. The true lumen expanded in circumference and cross-sectional area along the aortic arch, descending thoracic aorta, and abdominal aorta. The true lumen curvature at the aortic arch was reduced, and so was the maximum curvature at the descending thoracic aorta. This is likely the result of the structural influence of the thoracic endograft (straightening and radial expansion) along the aorta with TBAD. Also, additional expansion was introduced by the bare metal stent at the abdominal aorta. Notable increase in diameter and volume in the aortic true lumen from pre- to postoperative states is consistent with the previous studies monitoring the patients with conventional TEVAR and TEVAR with PETTICOAT techniques.^10^, ^11^, ^12^

The whole aorta, on the other hand, did not exhibit dramatic changes in the arclength or curvature. The cross-sectional geometric features (circumference and cross-sectional area) depicted the expansion of the whole aorta, primarily at the descending thoracic aorta, from pre- to post-TEVAR. Significant expansion of the true lumen likely led to subsequent expansion of the whole aorta in both circumference and cross-sectional area. The false lumen area at the abdominal aorta remained stable, whereas the false lumen area at the aortic arch and descending thoracic aorta exhibited insignificant shrinkage of 11% from pre- to post-TEVAR. Since 14 of 20 patients demonstrated partial or complete false lumen thrombosis during the follow-up period, it seems that the false lumen was thrombosed but not shrunken during this study's time interval between the pre- and post-TEVAR CT. This false lumen thrombosis rate is consistent with VIRTUE Registry (45%-55% at 1 month)^3^ and with Qiu et al.^25^

Several studies compared conventional TEVAR with TEVAR with PETTICOAT to examine the value of the PETTICOAT technique. Matsuoka et al^10^ reported the lower incidence of abdominal aneurysmal degeneration in PETTICOAT patients compared with conventional patients with TEVAR (8% vs 38%), and a lower risk of aortic erosion in patients with PETTICOAT compared with conventional patients with TEVAR (0% vs 33%). Qiu et al^25^ reported that the aorta-related mortality rate and complete false lumen thrombosis rate are similar between patients with PETTICOAT and conventional patients with TEVAR. Still, patients with PETTICOAT exhibited a significantly lower reintervention rate and a lower stent-graft-induced new-entry rate (*P* < .005). Niklas et al^26^ reported similar findings, a lower rate of secondary endovascular reintervention (*P* < .05) with no significant difference in mortality. Overall, the PETTICOAT technique can contribute to favorable aortic remodeling in patients for whom conventional TEVAR may not be enough to isolate the false lumen. Based on the geometric findings of the present study and existing comparative literature,^10^^,^^25^^,^^26^ TEVAR with PETTICOAT may be particularly beneficial for complicated patients with TBAD at elevated risk of distal false lumen aneurysmal degeneration. However, prospective comparative data are in demand to define optimal patient selection.

There are several limitations in this study: the subject cohort size is relatively small. The subjects were identified by the utilization of specific devices and PETTICOAT cases, but overall institutional TEVAR volume was not systematically captured. In addition, the heterogeneous distribution of dissection acuity limits acuity-specific conclusions; a subgroup analysis was performed, but was underpowered given the small sample sizes per group. Race and ethnicity data were not collected during enrollment and could not be reported; this limits the generalizability of findings across demographic groups. Variation in the time interval between preintervention and postintervention is another factor limiting our ability to conclude on aortic remodeling, as the geometric adaptation of the aorta to the intervention may vary over time. A lack of dynamic data may influence the interpretation of the geometric comparison, given the dynamic nature of the proximal thoracic aorta. All CT acquisitions were nongated, which may introduce variability in curvature measurements due to cardiac motion; however, such variability is expected to be random rather than systematic, and the reproducibility of these metrics using nongated protocols has been previously validated.^21^^,^^22^ Finally, the absence of a concurrent TEVAR-alone control group precludes direct attribution of the observed remodeling to the PETTICOAT component specifically; a comparative study of TEVAR alone vs TEVAR with PETTICOAT is needed to isolate the contribution of bare metal stent augmentation to aortic geometric remodeling.

In conclusion, this study provides a foundation for evaluating the geometric changes induced by TEVAR and PETTICOAT techniques. This hybrid procedure results in noticeable geometric changes of the dissected aorta at the aortic arch and thoracoabdominal aorta. The structural influence of the thoracic endograft induced straightening and radial expansion of the true lumen it covered. The bare metal stent augmented the cross-sections of the dissected abdominal aorta. TEVAR and PETTICOAT techniques are effective in opening the true lumen, with accompanying false lumen thrombosis, although false lumen cross-sectional remodeling was not significant. Long-term data analysis is needed to better understand aortic remodeling in the true and false lumens.

## Author Contributions

Conception and design: G-YS, JB, CC, BU

Analysis and interpretation: G-YS, JB, SR, KN

Data collection: G-YS, BU

Writing the article: G-YS, KN

Critical revision of the article: G-YS, JB, SR, CC, BU

Final approval of the article: G-YS, JB, SR, KN, CC, BU

Statistical analysis: G-YS

Obtained funding: G-YS

Overall responsibility: G-YS

## Funding

This work was supported by the 10.13039/100000002National Institutes of Health Grant R16HL178878 (to G.-Y.S.).

## Disclosures

None.
